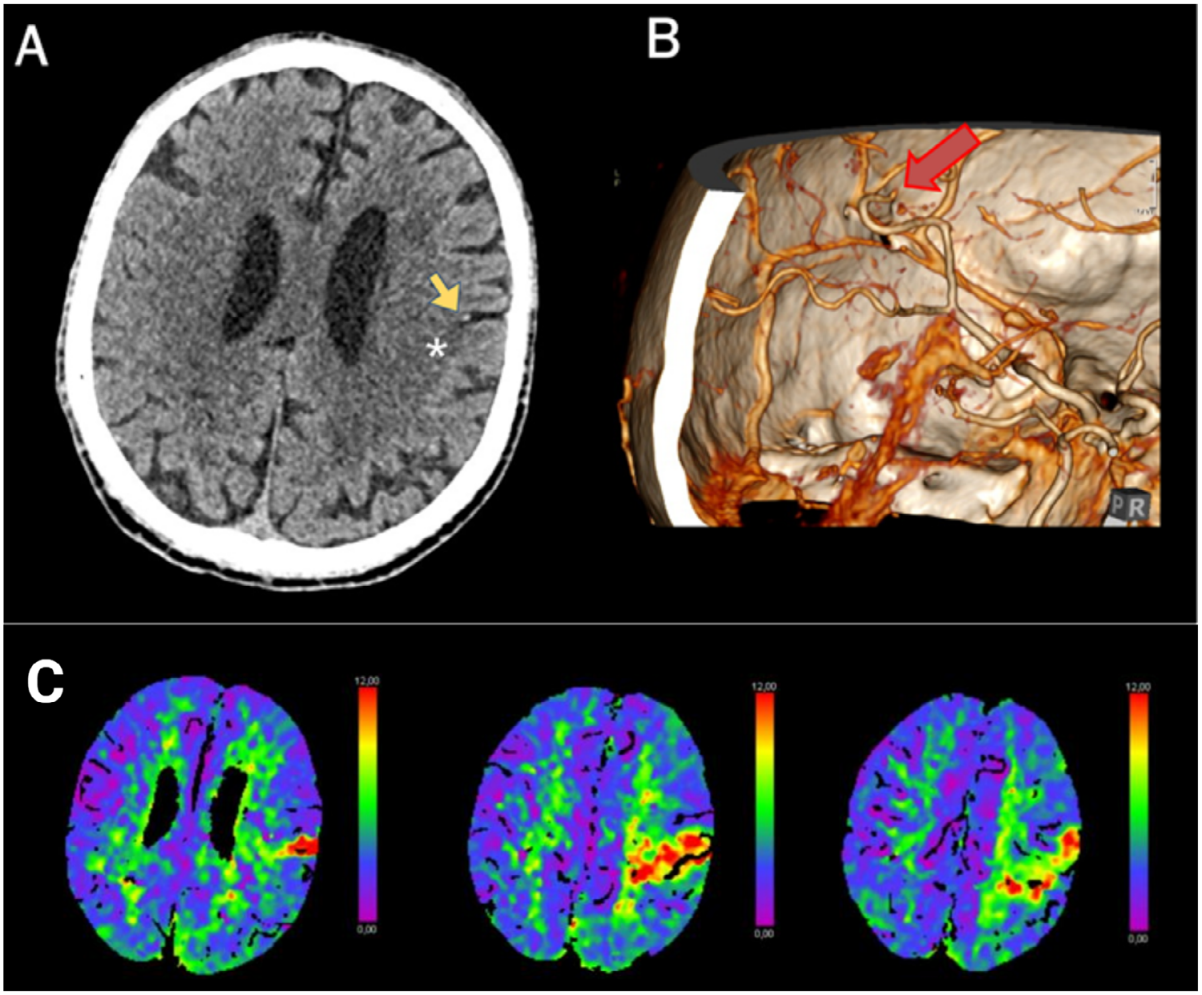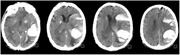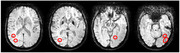# Challenges in Managing Cerebral Amyloid Angiopathy in Alzheimer's Disease: Insights from a Case of Down Syndrome‐associated Alzheimer Disease (DSAD)

**DOI:** 10.1002/alz70856_100856

**Published:** 2025-12-25

**Authors:** Lucía Maure‐Blesa, Maria Borrell Pichot, Íñigo Rodríguez‐Baz, Patricia Carbonell Fernández, Javier Arranz, José Enrique Arriola‐Infante, Mateus Rozalem Aranha, Alexandre Bejanin, Isabel Barroeta, Juan Fortea, Pol Camps‐Renom, Maria Carmona‐Iragui

**Affiliations:** ^1^ Sant Pau Memory Unit, Hospital de la Santa Creu i Sant Pau, Institut de Recerca Sant Pau ‐ Universitat Autònoma de Barcelona, Barcelona, Spain; ^2^ CIBERNED, Network Center for Biomedical Research in Neurodegenerative Diseases, National Institute of Health Carlos III, Madrid, Spain; ^3^ Hospital Sant Pau, Barcelona, Barcelona, Spain; ^4^ Center of Biomedical Investigation Network for Neurodegenerative Diseases (CIBERNED), Madrid, Spain; ^5^ Sant Pau Memory Unit, Hospital de la Santa Creu i Sant Pau, Biomedical Research Institute Sant Pau, Universitat Autònoma de Barcelona, Barcelona, Spain; ^6^ Sant Pau Memory Unit, Department of Neurology, Institut d’Investigacions Biomèdiques Sant Pau‐Hospital de Sant Pau, Universitat Autònoma de Barcelona, Barcelona, Spain; ^7^ Center for Biomedical Investigation Network for Neurodegenerative Diseases (CIBERNED), Madrid, Spain; ^8^ Barcelona Down Medical Center, Fundació Catalana Síndrome de Down, Barcelona, Spain; ^9^ Center for Biomedical Investigation Network for Neurodegenerative Diseases (CIBERNED), Madrid, Madrid, Spain; ^10^ Stroke Unit, Department of Neurology, Hospital de La Santa Creu I Sant Pau, IR Sant Pau, Barcelona, Spain, Barcelona, Barcelona, Spain

## Abstract

**Background:**

Cerebral amyloid angiopathy (CAA) is more frequently found in Down syndrome associated Alzheimer's disease (DSAD) than in sporadic forms of Alzheimer's disease (AD). Although the pathophysiological process starts before, in Down syndrome (DS), neuroimaging features of CAA can be detected from the early 30s and progress with age. CAA poses significant challenges in clinical management, particularly in the context of acute cerebrovascular events occurring in adults with DSAD. We present a case that reveals the limited evidence to guide treatment decisions in acute ischemic strokes where DSAD and CAA overlap.

**Methods:**

A 58‐year‐old male with DS and prodromal AD presented in 2024 with acute aphasia, right hemianopsia, and right hemiparesis. His medical history included epilepsy, asymptomatic neutropenia, and a pacemaker for sinus bradycardia. Non acute lesions were evident in the urgent brain CT scan (Figure 1A), while CT angiography revealed a distal M2/M3 occlusion in the left middle cerebral artery (Figure 1B). Perfusion CT demonstrated an ischemic area in the left frontal region, leading to the diagnosis of an acute ischemic stroke in the left MCA territory (Figure 1), and meeting criteria for thrombolysis, so alteplase was administered.

**Results:**

During alteplase administration, the patient experienced clinical deterioration. A subsequent CT scan evidenced an intracranial hemorrhagic transformation (Figure 2). Thereafter, an MRI from 2018 was reviewed, demonstrating lobar microbleeds consistent with suspected CAA, although no prior diagnosis had been established (Figure 3). Despite significant hemorrhage, the patient demonstrated unexpected clinical improvement.

**Conclusions:**

CAA is particularly common in DSAD due to the genetic foundation of AD, which accelerates beta‐amyloid pathology in both the brain parenchyma and blood vessels. This case underscores a significant gap in the literature concerning the coexistence of CAA in DSAD and acute ischemic stroke. Presently, there is no established evidence to guide management in such cases, and this issue is likely underreported due to limited data on reperfusion therapy for stroke in individuals with DS. The genetic characteristics of DSAD present a unique opportunity to explore the progression and interplay of these conditions, potentially providing valuable insights to inform treatment strategies and enhance outcomes for all patients.